# Supplementary material for: Chromothripsis during telomere crisis is independent of NHEJ, and consistent with a replicative origin
Source: Genome Res. 2019 May;29(5):737–49. doi: 10.1101/gr.240705.118 (PMC6499312; doi:10.1101/gr.240705.118)
Supplement: Supplemental Material [file supp_gr.240705.118_Supplemental_file_1.zip › contigs/annotated_contigs/DB54/contig.2.DB54_length_448_mean_cov_6.9375.docx]

**DB54_length_448_mean_cov_6.9375**

```
AGGAGTTGGTGCCACGCAGAAAGGGGCTGACCGCATGGTTGAAGAGTCTTAGCTGAGAGGTATGTTTATATTCTTGTCAAAGGAAATTT
 >chr4:2911885-2912013 + E=2e-65
CCAGAACTGTCTCTTTCACTTGTCACACCTTCATTGAGA|CCTTGTCTCAAAAATAAAT|AAATAAATAAATAAATAAATAAATAAAAT
                                                           >chr4:2697756-2698057 + E=9e
ACATTAATCAGGATTTGTAATTGTTTCCTTTTCAGTTTAAAAAATCCACTTATGTGCATAGCTATACATGCTTTATCAGGTTTTTCATG
-170
AATATGTAAAATTTGAGAGTAGGTATCAAAATGCCACTTTTGGGTGACTTGCTGATAGGTTTTTCTCTTTTTCTGCTGGTTGTTGTATT

TCCTTATTTTTCACAACAAATATATACTACTTGAGTAATAAAACAGTTTATTTTTGGAGGTGTGGTCTGAAATTTATTATATTCAAAAT

TTTTA
```
